# Retinal Vascular Tortuosity and Diameter Associations with Adiposity and Components of Body Composition

**DOI:** 10.1002/oby.22885

**Published:** 2020-07-29

**Authors:** Robyn J. Tapp, Christopher G. Owen, Sarah A. Barman, Roshan A. Welikala, Paul J. Foster, Peter H. Whincup, David P. Strachan, Alicja R. Rudnicka, for the UK Biobank Eye, Vision Consortium

**Affiliations:** 1 Population Health Research Institute, St George’s, University of London, London, UK; 2 Melbourne School of Population and Global Health, The University of Melbourne, Melbourne, Australia; 3 Faculty of Science, Engineering and Computing, Kingston University, Surrey, UK; 4 Integrative Epidemiology Research Group, UCL Institute of Ophthalmology, London, UK; 5 NIHR Biomedical Research Centre at Moorfields Eye Hospital, London, UK

## Abstract

**Objective:**

The aim of this study was to assess whether adiposity or body composition relates to microvascular characteristics of the retina, indicative of cardiometabolic function.

**Methods:**

A fully automated QUARTZ software processed retinal images from 68,550 UK Biobank participants (aged 40-69 years). Differences in retinal vessel diameter and tortuosity with body composition measures from the Tanita analyzer were obtained by using multilevel regression analyses adjusted for age, sex, ethnicity, clinic, smoking, and Townsend deprivation index.

**Results:**

Venular tortuosity and diameter increased by approximately 2% (*P* < 10^–300^) and 0.6 μm (*P* < 10^–6^), respectively, per SD increase in BMI, waist circumference index, waist-hip ratio, total body fat mass index, and fat-free mass index (FFMI). Venular associations with adiposity persisted after adjustment for FFMI, whereas associations with FFMI were weakened by FMI adjustment. Arteriolar diameter (not tortuosity) narrowing with FFMI was independent of adiposity (–0.6 μm; –0.7 to –0.4 μm per SD increment of FFMI), while adiposity associations with arteriolar diameter were largely nonsignificant after adjustment for FFMI.

**Conclusions:**

This demonstrates, on an unprecedented scale, that venular tortuosity and diameter are more strongly associated with adiposity, whereas arteriolar diameter relates more strongly to fat-free mass. Different attributes of the retinal microvasculature may reflect distinct roles of body composition and fatness on the cardiometabolic system.

## Introduction

The global rise in obesity is a critical public health problem. High levels of body fatness have led to a marked rise in type 2 diabetes and an increased risk for cardiovascular disease (CVD) (1,2), with close to 70% of deaths related to high BMI caused by CVD (1). Body composition, particularly central adiposity, is thought to play a major role in this increased risk beyond general adiposity as measured by BMI (2), and this has been linked to an increased risk for mortality (3). It has previously been shown that adult body fat is more strongly associated with type 2 diabetes and CVD than BMI alone (4–6), as BMI is unable to distinguish fat distribution, an important issue, best highlighted among normal weight individuals who have an increased visceral fat mass (FM) and greater cardiometabolic risk (7).

The microvasculature has increasingly been recognized as an early determinant of both obesity and CVD risk (8). In a population-based study of adults (aged 44-85 years) from Wisconsin, increased venular diameter was associated with an increased risk for developing obesity over a 15-year period (8). Retinal arteriolar narrowing with higher BMI was reported among healthy individuals (9), and a systematic review of population-based studies found retinal arteriolar narrowing and venular widening with higher BMI (10). Though a large body of research has observed that higher BMI is associated with wider and more tortuous venules (11) and narrower and less tortuous arterioles (10), limited research has assessed other body composition measures (including indices quantifying total FM and fat-free mass [FFM]) in adulthood, and very few population-based studies have assessed the associations with retinal tortuosity (11,12).

Measurement of body composition beyond BMI provides a range of morphometric indices (including both body FM and lean mass), which may assist in furthering our understanding of the mechanisms linking increased adiposity with adverse systemic changes in the microvasculature (13). To date, these associations have not been examined at scale. The retinal microvasculature can be assessed non-invasively and it provides a general assessment of the microvasculature throughout the body (14). Given the strong associations between greater venular diameter with incident diabetes (15) and CVD events (16) (myocardial infarction or stroke), understanding the early associations between the retinal microvasculature and body composition important.

Though semiautomated, noninvasive assessment of the retinal microvasculature has been available for many years (17), it is only now that the first fully automated software is available for use at scale (Quantitative Analysis of Retinal Vessel Shape and Size [QUARTZ]) (18,19). This offers an unprecedented opportunity to examine the shape and form of association between adiposity markers with retinal vessel morphometry (including tortuosity, a measure that has not been well researched to date). The aim of this study was to examine the association of retinal vessel morphometry (diameters and tortuosity) with body composition measures and provide a comparative analysis of these associations.

## Methods

**Figure 1:**
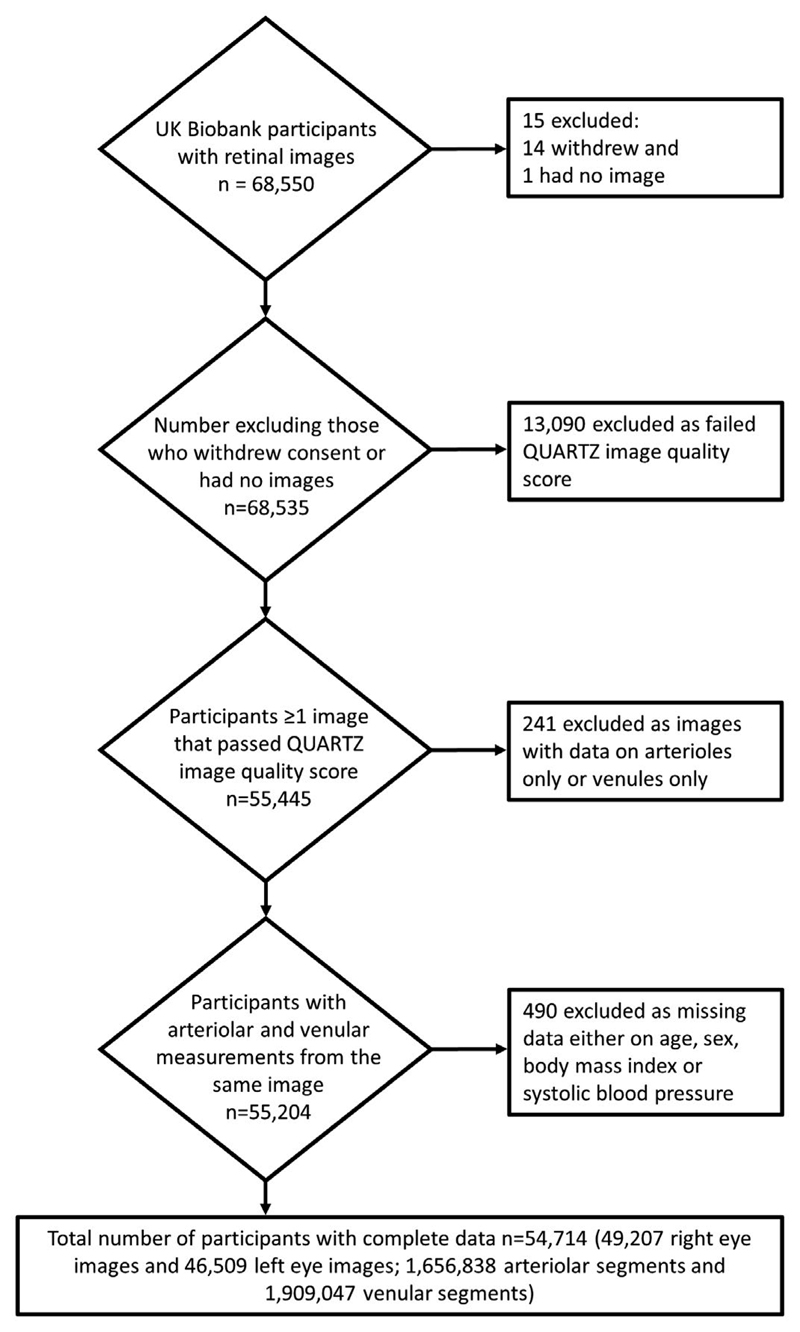
Flow diagram of participants included in the analyses.

The UK Biobank is a national research resource, offering the opportunity to investigate and develop techniques aimed at improving the prevention, diagnosis, and treatment of a wide range of noncommunicable diseases (20). The study recruited more than 500,000 people aged 40 to 69 years between 2006 to 2010 from across the United Kingdom. A subset of 68,550 participants had macular-centered retinal images taken (from one or both eyes). The design and methods in the UK Biobank Eye and Vision Consortium have recently been published (21). The eye and vision substudy has a very similar profile to the main UK Biobank study. For example, the mean BMI (a key variable of interest in this manuscript) was 27.4 (SD 4.8) in the full UK Biobank cohort and 27.4 (SD 4.8) in the eye and vision substudy. A flowchart of participants by exclusions from the UK Biobank eye study is shown in Figure 1. Color images were captured without mydriasis using a nonmydriatic fundus camera (Topcon 3D OCT-1000 Mk 2) with a 45° field of view and saved in PNG format with a resolution of 2,048 × 1,536 pixels.

### Physical examination

Height was measured to the nearest centimeter by using a Seca 202 stadiometer, and a Tanita BC-418MA body composition analyzerwas used to measure weight to the nearest 0.1 kg and assess body composition. BMI was calculated as weight (kilograms) divided by height (meters squared) and categorized according to the World Health Organization definitions into underweight (< 18.5), normal weight (18.5 to < 25), overweight (25 to <30), and obesity (≥ 30). Body size measures included fat percentage (F%), FM, and FFM both total and trunk (total F% represents the percent of body weight that is fat, whereas trunk fat % represents the percent of total fat that is estimated to be in the trunk). Waist circumference at the level of the umbilicus and hip circumference were measured by using a Wessex nonstretchable sprung tape measure. Waist circumference, FM, and FFM were indexed to height squared to provide body measures that were independent of height. Two blood pressure (BP) measurements were taken with an Omron HEM-705 IT automated electronic BP monitor while seated, at least 1minute apart (22), and the average of the two measures was used in the analyses.

### Retinal imaging and processing

**Figure 2:**
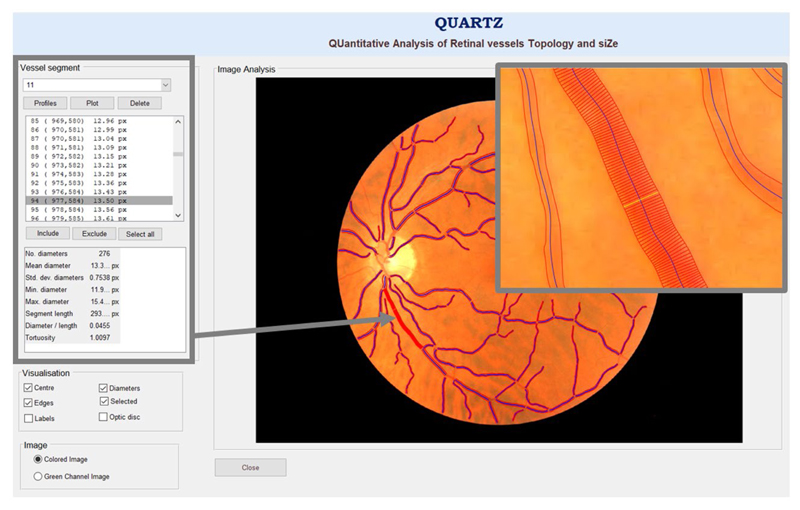
Quantitative analysis of retinal vessel topology and slze (QUARTZ) software.

Processing of retinal images was carried out using an automated computerized system (QUARTZ), as previously published in detail (23). In brief, the automated system distinguishes between right and left eyes (by optic disc localization) and venules and arterioles, identifies vessel segments, outputs centerline coordinates, and measures vessel width and angular change between vessel centerline coordinates, as well as provides further measures of tortuosity (Figure 2) (23). These measures were summarized as a mean width per image, weighted by segment length, for arterioles and venules separately for each image. The following image processing modules were all validated on a subset of 4,692 retinal images from a random sample of 2,346 UK Biobank participants: vessel segmentation, image quality score, optic disc detection, vessel width measurement, tortuosity measurement, and arteriolar venular recognition (19,23,24). The performance of the arteriole/venule recognition had detection rates of up to 96% for arterioles and 98% for venules when the automated probability of artery or vein was set to a cutoff of 0.8. An automated assessment of image quality was also made based on the segmented vasculature (24). The algorithm achieved a sensitivity of 95.3% and a specificity of 91.1% for the detection of inadequate images (19). A model eye was used to quantify the magnification characteristics of the Topcon 3D OCT-1000 Mk 2 fundus camera, allowing pixel dimensions of vessel diameter to be converted to real size (25).

### Other covariates

Age, sex, smoking status, medication use, and other sociodemographic characteristics were collected from a computerized questionnaire. Heart attack, stroke, and diabetes were determined by self-report. At a postcode level, neighborhood deprivation was expressed in terms of the Townsend deprivation index.

The UK Biobank study was reviewed and approved by the Northwest Region National Health Service research ethics committee.

### Statistical analysis

Data analysis were performed with Stata 15.0 IC. Segmentwise weighted mean widths and tortuosity were used to provide a measure for venules and arterioles separately for each eye. Histograms of retinal vessel widths showed normal distributions, while measures of tortuosity were positively skewed and log-transformed. Data missing on categorical variables were included as an additional category for each variable to minimize data loss. Multilevel linear regression models adjusting for age, sex, ethnicity, and UK Biobank center were used to examine associations of body composition measures with retinal vessel outcomes, allowing for repeated measures of vessel indices within the same person (model 1). Model 2 provided further adjustment for the confounding effects of smoking and Townsend deprivation index, and model 3 excluded those who had heart attack, stroke, or diabetes; were on treatment for hypertension; or answered unknown or declined to answer. Body composition measures were modeled as *z* scores in the regression models, and all coefficients represent the absolute difference in vessel width and percentage difference in tortuosity per 1-SD increase in body composition measure. To address concerns over sexual dimorphism in the associations between body composition and retinal vessel morphometry, we formally examined interactions with sex (*P* value for interaction was set to < 0.01 for statistical significance given the large sample size of UK Biobank) and whether associations could be presented for males and females combined. The independence of associations with body composition measures reflecting adiposity were further examined by adjustment for FFM index (FFMI).

## Results

The characteristics of the population overall and by BMI category are shown in Table 1. A total of 54,714 participants (1,656,838 arterioles and 1,909,047 venules) were included in these analyses.

**Figure 3:**
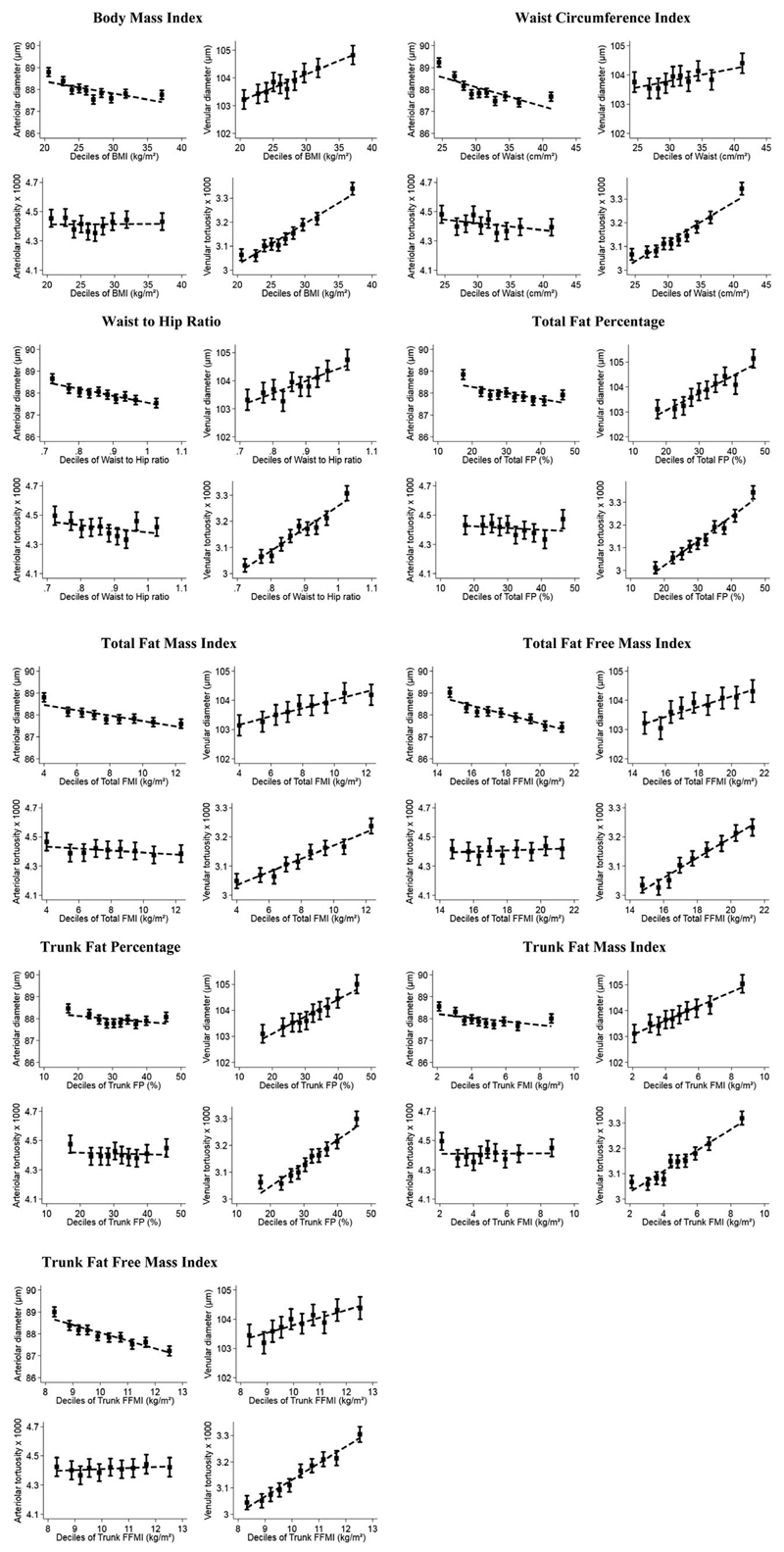
Adjusted mean retinal vessel width and tortuosity by deciles of age and body composition measures. Adjusted means (solid square symbols), 95% CIs (solid vertical error bars), and regression line (dotted line) are from a multilevel model allowing for age and sex as fixed effects and repeated retinal vessel measures within each person.

Figure 3 shows the association of retinal diameters and tortuosity with each body composition marker in deciles, adjusted for age and sex as fixed effects and a random effect for person. Venular diameter and tortuosity showed strong positive linear associations with each decile increase in body composition measures. Arteriolar diameter showed a negative linear association with higher BMI, waist circumference index, waist-hip ratio, F%, fat mass index (FMI), and FFMI for each region (including total and trunk measures). No clear associations were evident between arteriolar tortuosity and body composition measures.

### Associations with age and sex

Although females had, on average, more tortuous arterioles (4.4%; 95% CI: 3.4% to 5.4%) and venules (1.5%; 95% CI: 0.9% to 2.0%) and narrower arterioles (–0.2 μm; 95% CI: –0.3 to –0.05 μm) and venules (–0.8 μm; 95% CI: –1.0 to –0.5 μm), these sex differences were largely related to height difference between males and females (Table 2, model 3). Formal tests of interaction for the associations of retinal venular measures with age and sex (females vs. males adjusted for ethnicity and UK Biobank center) were not statistically significant for venular tortuosity (*P* = 0.27 for interaction) or venular diameter (*P* = 0.91 for interaction). For each decade rise in age, venular tortuosity increased by 2.5% (95% CI: 2.1% to 2.8%) and venules widened by 1.0 μm (95% CI: 0.9 μm to 1.2 μm; Table 2, model 1). Adjustment for confounders in model 2 and removal of those with diabetes, heart attack, or stroke or those on medication for BP (model 3) had no impact on the associations observed.

Formal tests of interaction for the associations of retinal arteriolar measures with age and sex were statistically significant for arteriolar tortuosity (in males, 1.6%; 95% CI: 0.8%-2.3% per decade rise in age; in females, 3.1% 95% CI: 2.4%-3.8% per decade rise in age; *P* = 0.004 for interaction) and not for arteriolar diameter (*P* = 0.206 for interaction), adjusted for ethnicity and UK Biobank center as fixed effects and random effect for person. Arteriolar tortuosity association with age was in the same direction but slightly steeper in females compared with males.

### Sex differences: body composition and retinal vessel measures

Formal tests of interaction for the association of body size measures and sex (from multilevel model adjusted for age, ethnicity, and UK Biobank center) for each microvascular measure were not statistically significant in all cases (*P* > 0.01) except for the following associations with arteriolar diameter only: BMI (males, –0.39 μm per SD kg/m^2^; females, –0.22 μm per SD kg/m^2^; *P* = 0.013 for interaction), waist circumference index (males, –0.69 μm per SD cm/m^2^; females, –0.41 μm per SD cm/m^2^; *P* = 4.6 × 10^–8^ for interaction), and similar findings for F% and FMI. For each of the significant interactions observed, the associations were marginally steeper in males compared with females, but the overall difference in slopes was minimal. Hence, associations presented in Tables 2 and 3 are for males and females combined.

### Retinal vessel tortuosity associations with body composition

Increased venular tortuosity was associated with higher BMI, waist circumference index, waist-hip ratio, F%, FMI, and FFMI (for each region, total and trunk; Table 2, model 1), and most of these associations were highly statistically significant with *P* < 1 × 10^–300^. The strongest association was observed between venular tortuosity and total FFMI, followed by total F%. For each SD increase in total FFMI, venular tortuosity increased by 2.8% (95% CI: 2.4%-3.2%; Table 2, model 1) and total F% by 2.6% (95% CI: 2.2%-2.9%). The associations observed were unaffected by adjustment for confounders (model 2) or removal of those who reported diabetes, heart attack, or stroke events (model 3). No association between arteriolar tortuosity and body composition measures was observed. The association of venular tortuosity with each measure of body composition was independent of venular diameter (data not shown).

### Retinal vessel diameter associations with body composition

Narrower arteriolar diameter was associated with higher BMI, waist circumference index, waist-hip ratio, F%, FMI, and FFMI (for total and trunk) (Table 3, model 1). The strongest associations were observed between arteriolar diameter and FFMI and waist circumference index. Additional adjustment for potential confounding factors (smoking and Townsend deprivation index) had no impact on the associations (model 2) . The associations with each adiposity measure were unaffected by removal of those with heart attack, stroke, or diabetes; those on treatment for hypertension; or those who answered unknown or declined to answer (model 3).

Wider venular diameters were associated with higher levels of all adiposity and body size measures after adjustment for age, sex, UK Biobank center, and ethnicity (Table 3, model 1). The strongest association was observed between venular diameter and total F%. For each SD increase in total F%, venular diameter widened by 0.7 μm (95% CI: 0.6-0.8 μm; Table 3, model 1). Additional adjustment for potential confounding factors (smoking and Townsend deprivation index) had no impact on the associations (Table 3, model 2). The associations in model 2 were unaffected by removal of those with heart attack, stroke, or diabetes; those on on treatment for hypertension; or those who answered unknown or declined to answer (Table 3, model 3).

### Models mutually adjusting body composition measures for FFMI

**Figure 4:**
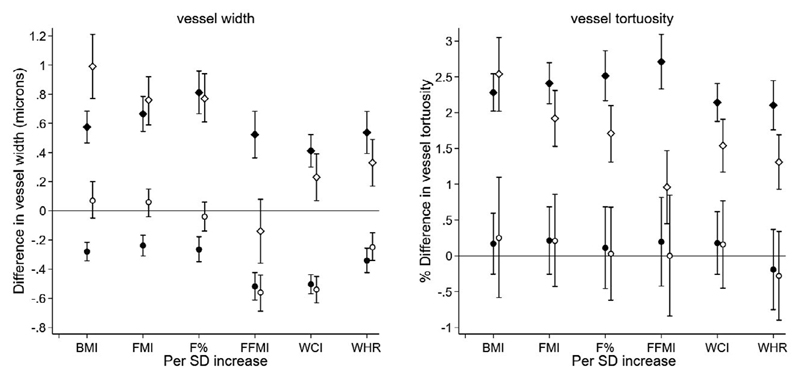
Adjusted mean differences with 95% CIs (error bars) from multilevel regression model adjusted for age, sex, ethnicity, UK Biobank center, smoking, Townsend deprivation index, and random effect for person; solid diamond symbols are associations in venules, and solid circular symbols are for arterioles (model 2 in Tables 2 and 3). Additional adjustment for fat-free mass index (FFMI; adjusted for fat mass index [FMI]) is shown in open diamond symbols for venules and open circles for arterioles. Differences are per SD rise in height-standardized body composition measures. BMI, body mass index; F%, total fat%; WCI, waist circumference index; WHR, waist hip ratio.

Findings from regression models mutually adjusting for total FMI and total FFMI are shown in Figure 4 and Supporting Information Table S1. For arteriolar diameter the inverse association with total FFMI remains stable (–0.6 μm; 95% CI: –0.7 to 0.4 μm; *P* = 6.10 × 10^–18^ per SD rise in total FFMI) after adjustment for total FMI, whereas the inverse association with total FMI is attenuated to the null and no longer statistically significant. For venular diameter, the positive association with total FMI is stable after adjustment for total FFMI, but the association with total FFMI is attenuated to the null and not statistically significant. The positive associations of total FFMI and total FMI with venular tortuosity persist after mutual adjustment, but the association with total FMI is stronger.

For the other body composition measures, associations with arteriolar diameter were predominantly a null (except waist circumference and waist-hip ratio) after adjustment for total FFMI and associations with total FFMI prevailed (Figure 3 and Supporting Information Table S1). In general, associations between venular measures and the other body composition measures were independent of adjustment for FFMI (Figure 3 and Supporting Information Table S1); although associations of FFMI with venular tortuosity were independent of adjustment for the other body size measures, FFMI associations with venular diameter were confounded by body fat measures.

## Discussion

This is the first study to assess the association of different body composition measures with the retinal vessel morphometry at scale. Each measure of body composition showed a graded association with vessel morphometry measures (diameters and tortuosity), with the exception of arteriolar tortuosity, which showed no evidence of association. The associations held after adjustment and removal of those with diabetes, CVD morbidity, or hypertension. These observations show definitive associations between body composition and retinal diameters and provide evidence of novel associations with retinal tortuosity. Moreover, we show that positive adiposity associations with venular diameter and tortuosity prevail after adjustment for FFMI, and FFMI associations with venular tortuosity are independent of adiposity measures; however, inverse FFMI associations with arteriolar width persist after adjustment for FMI, suggesting different effects of body size and composition on retinal vasculature.

### Retinal tortuosity

In the present study, we showed a positive association between higher venular tortuosity with increased adiposity (BMI, waist circumference index, waist-hip ratio, F%, FMI [both total and trunk], and lean mass). Total F%, FMI, and FFMI each showed a similar strong association with venular tortuosity, as did BMI. Earlier work has only examined associations with BMI and not with other more detailed measures of body size (9,12). Previous studies have been small (with samples sizes ranging from 167 to 5,947) with some underpowered to detect associations. Associations detected have gone in both directions, with reduction of tortuosity with increased BMI in some and an increase of tortuosity or no association in others (9,11,12,26). In a study by Cheung et al. (12) (*n* = 2,915), higher BMI was associated with less tortuous retinal arterioles, with no association evident for venules, the exact opposite of the present study that showed no association with arteriolar tortuosity and a strong positive association with venular tortuosity. It is possible that the sample size in the study by Cheung et al. (12) was too small to precisely quantify the associations that are subtle. In addition, the manual approach for delineating or selecting vessels near the optic disc only for measurement is likely to incur a higher degree of measurement error. East Asians generally have more myopia and a higher degree of myopia. Therefore, axial elongation of the eye will be greater, resulting in a more stretched retina; mechanically, if the retina is stretched, the vessels will as a result be straighter which may limit the potential of changes in vessels in relation to adiposity. In contrast, a study assessing a healthy cohort of adults aged 45 to 75 years showed no association between BMI and tortuosity (either arteriolar or venular) (9), as was the case for a study assessing a population with diabetes (type 1 and type 2) aged 18 to 70 years (26). In the most recent study to assess the association of retinal tortuosity with BMI in older adults (mean age 68 years), the European Prospective Investigation into Cancer-Norfolk Eye study showed increased venular tortuosity was associated with higher BMI (2.5%; 95% CI: 1.7%-3.3% per 5 kg/m^2^), with no evidence of an association for arteriolar tortuosity (11), in agreement with this report. Of interest, arteriolar tortuosity has previously been shown to have a high heritability factor compared with venular tortuosity, suggesting venules are more influenced by systemic changes than arterioles (27). In a study by Kirin et al. (27) the heritability for arteriolar tortuosity was 55%, and for venular tortuosity, it was 21%.

The second novel finding of the present study that body composition measures (total: F%, FMI, and FFMI) were more or as strongly associated with venular tortuosity as BMI, waist circumference index, or waist-hip ratio (Table 2) suggests that venular tortuosity may be affected by overall FM. This finding supports earlier research that had suggested body composition measures (F%, FMI) may better reflect overall body fat distribution than BMI and, therefore, relate more strongly to changes in the microvasculature (28,29). The independent association of venular tortuosity with FFMI (adjusted separately for each body composition measure) suggests that not only markers of adiposity are of potential importance and warrants further investigation.

### Retinal diameters

The marked difference in associations between adiposity and retinal diameters in arterioles and venules observed in the present study have been reported in previous studies (9,10,11,30,31). Higher BMI and inflammation have generally been associated with wider venules and higher BP and (with less clear results) BMI with arteriolar narrowing (10), consistent with the findings of the present study. Our previous report based on European Prospective Investigation into Cancer-Norfolk Eye study showed very similar increased venular diameter with higher BMI (0.72 μm; 95% CI: 0.04-1.03 μm per 5 kg/m^2^) but with no evidence of an association for arteriolar diameter (11). BMI, F%, and FMI were more strongly associated with venular widening in the present study. While stronger associations were evident for F% and FMI than for BMI, these differences were modest. Waist circumference, waist-hip ratio, and FFMI were more strongly associated with arteriolar narrowing. This is the first adult study to assess these additional measures of body composition (i.e., waist-hip ratio, F%, FMI, and FFMI); hence, the observation that these body composition associations with retinal vessel diameters vary by measure has not previously been assessed. Studies in children and adolescence have shown similar results and are consistent with the findings of the present study (28). In a study by Xiao et al. (28) (*n* = 731), young people aged 12 to 19 years with higher total FM, total FMI, trunk F%, triceps skin fold thickness, and BMI had wider venular diameter and narrower arteriolar diameter. A similar association was observed in the Avon Longitudinal Study of Parents and Children, in which increased FM and BMI at age 11 years were associated with wider venular diameter; however, no association was evident for arteriolar diameter (31). By modeling FFMI with each of the body composition measures, we have shown in the present study that the associations between each body composition measure (except waist circumference) and arteriolar diameter were confounded by FFMI (a measure of body build) and that FFMI remained independently associated with arteriolar diameter. This finding needs to be further explored in longitudinal data for which causality can be determined.

### Mechanistic pathways and clinical implications

While the mechanisms linking different body composition measures with changes in retinal diameters and tortuosity are yet to be fully established, adipose tissue secretes a range of adipocytokines that impact metabolic and CVD function (31). Adiposity in particular is associated with an increased expression of endothelial nicotinamide adenine dinucleotide phosphate oxidase and higher levels of oxidative stress (32). Research has shown venular widening is more evident among those with high inflammatory and endothelial dysfunction markers (33,34), an area that warrants further investigation, particularly for tortuosity. Studies have shown that changes in the systemic microvasculature are associated with increased adiposity (27). Of great interest, findings from our recent work suggest obesity-related microvascular changes are reversible after bariatric surgery–induced weight loss, suggesting plasticity of the human microvasculature early in the disease course (35).

Prospective studies have shown utility of retinal vessel diameter predicting future disease. Meta-analyses have shown that per SD decline in arteriolar diameter and per SD rise in venular diameter, the risk of stroke, death, hypertension, type 2 diabetes mellitus, and coronary heart disease increases by approximately 10% to 20% (36,37,38). However, evidence of prospective associations with tortuosity have not been published. In some instances, our cross-sectional associations with tortuosity are stronger than with vessel diameter; hence, we expect measure of tortuosity to be predictive of incident cardiovascular disease and type 2 diabetes mellitus.

### Study strengths and limitations

The strengths of the current study include its large sample size of over 3.5 million vessel segments from 50,000 participants. The breadth of adiposity and body composition measures, unprecedented on this scale, allow consistency of associations across different measures and mutual independence to be assessed. The current study has some limitations. It was cross-sectional, and, therefore, it was not possible to draw firm conclusions on causal associations, as longitudinal analyses would be required. Although the current study did not include all Biobank study participants, an issue if one were attempting to determine prevalence, this study was focused on assessing association and would be unaffected using the smaller sample of over 50,000 participants. Further longitudinal follow-up is warranted using the fully automated quantification (QUARTZ) to determine at scale the causality of the association.

## Conclusion

These observations highlight novel and strong associations between retinal venular tortuosity and body composition measures, the independent impact of adiposity markers from FFMI, and they provide confirmatory evidence of diameter associations and raise the spectra that associations with arteriolar diameter and general measures of body adiposity are confounded by lean mass. Importantly, the study is unique in showing associations with more detailed measures of body composition, including assessments of fat and FFMI. However, further longitudinal research is needed to fully elicit causal associations, particularly with novel measures of vessel tortuosity, and determine their usefulness in cardiometabolic disease risk prediction.**O**

## Supplementary Material

Supplementary Table S1

## Figures and Tables

**Table 1:** Characteristics of population by BMI category

	Total	Underweight	Healthy weight	Overweight	Obese
	54,714	252	17,943	23,403	13,116
**Age (y)**	56.1 (8.2)	55.4 (7.9)	55.3 (8.3)	56.6 (8.2)	56.4 (8.0)
**Gender (% female)**	55	80	67	47	53
**Ethnicity (%)**					
**White**	90.9	88.1	91.8	91.0	89.4
**Black**	2.9	1.2	1.5	2.8	4.9
**Asian**	2.8	4.0	2.9	3.0	2.3
**Other**	2.8	5.2	3.2	2.5	2.5
**Unknown/didn’t answer**	0.7	1.6	0.5	0.7	0.9
**Smoking (%)**					
**Never smoker**	55.9	59.1	59.8	54.8	52.5
**Occasionally**	2.9	2.8	2.8	3.1	2.8
**Ex-smoker**	33.9	23.4	29.7	35.0	37.7
**Current smoker**	6.7	14.3	7.3	6.5	6.2
**Prefer not to say/missing**	0.6	0.4	0.4	0.6	0.8
**Quartiles of Townsend deprivation index (%)**					
**< –3.4**	25.1	21.0	26.2	25.9	22.2
**–3.4 to –1.6**	25.3	21.0	25.0	26.4	23.7
**–1.7 to 0.8**	25.0	20.6	25.5	24.8	24.9
**0.8**	24.5	36.9	23.2	22.8	29.0
**Missing**	0.1	0.4	0.1	0.1	0.2
**Systolic BP (mmHg)**	136.5 (18.3)	128.2 (20.9)	131.6 (18.5)	138.0 (17.8)	140.9 (17.3)
**Diastolic BP (mmHg)**	81.7 (10.0)	75.3 (10.6)	78.1 (9.7)	82.4 (9.6)	85.4 (9.6)
**BMI (kg/m^2^)**	27.3 (4.7)	17.6 (0.8)	22.7 (1.5)	27.2 (1.4)	33.8 (3.8)
**Waist circumference (cm)**	89.8 (13.4)	66.5 (6.2)	78.2 (8.1)	90.7 (8.4)	104.5 (11.0)
**Waist-hip ratio**	0.9 (0.1)	0.8 (0.1)	0.8 (0.1)	0.9 (0.1)	0.9 (0.1)
**Total fat %**	31.5 (8.5)	18.5 (5.3)	27.0 (6.9)	31.2 (7.4)	38.4 (7.9)
**Total fat mass (kg)**	24.8 (9.5)	9.4 (2.4)	17.2 (4.4)	24.2 (5.1)	36.6 (9.1)
**Total fat free mass (kg)**	53.1 (11.5)	40.0 (6.0)	47.2 (8.8)	54.3 (10.7)	59.1 (12.2)
**Trunk fat %**	0.0 (1.0)	–1.3 (1.4)	–0.3 (1.1)	0.2 (0.9)	0.0 (0.9)
**Trunk fat mass (kg)**	13.8 (5.2)	4.2 (1.8)	9.3 (2.7)	13.8 (2.8)	20.2 (4.5)
**Trunk fat free mass (kg)**	29.4 (5.9)	23.0 (3.4)	26.7 (4.8)	30.2 (5.7)	32.0 (6.2)
**Image quality**	0.9 (0.1)	0.9 (0.1)	0.9 (0.1)	0.9 (0.1)	0.9 (0.1)
**Arteriolar width (μm)**	88.0 (7.8)	89.3 (7.9)	88.4 (7.6)	87.8 (7.8)	87.8 (8.0)
**Venular width (μm)**	103.9 (13.1)	103.0 (12.1)	103.3 (12.9)	104.0 (13.2)	104.7 (13.4)
**Arteriolar tortuosity (x 1,000)**	4.4 (1.6)	4.6 (1.7)	4.4 (1.7)	4.4 (1.6)	4.5 (1.0)
**Venular tortuosity (x 1,000)**	3.2 (1.4)	3.1 (1.4)	3.1 (1.3)	3.1 (1.4)	3.3 (1.0)
**Heart attack (%)**	1.8	1.2	1.0	1.9	3.0
**Stroke (%)**	1.1	1.6	0.9	1.1	1.6
**Diabetes (%)**	4.9	1.6	2.0	4.1	10.5

Data given as mean (SD) or percent. Data missing on categorical variables included as additional category for each variable to minimize data loss. Data missing on the following continuous variables: waist circumference (*n* = 16), waist-hip ratio (*n* = 20), body fat% (*n* = 1,288), body fat mass (*n* = 1,356), body fat-free mass (*n* = 1,259), trunk fat % (*n* = 1,255), trunk fat mass (*n* = 1,255), and trunk fat-free mass (*n* = 1,255).

**Table 2:** Percentage difference in vessel tortuosity with age, sex, and per standard deviation increase in body composition measures

Risk marker	Percentage difference in arteriolar tortuosity (95% CI)						Percentage difference in venular tortuosity (95% CI)					
	Model 1		Model 2		Model 3		Model 1		Model 2		Model 3	
		*P*		*P*		*P*		*P*		*P*		*P*
**Age per decade**	2.4 (1.9, 2.9)	1.1E-19	2.5 (1.9, 3.0)	3.3E-20	2.3 (1.8, 2.9)	8.0E-16	2.5 (2.1, 2.8)	<1.0E-300	2.5 (2.2, 2.9)	<1.0E-300	2.4 (2.0, 2.7)	<1.0E-300
**Female versus male**	4.2 (3.4, 5.1)	1.3E-22	4.4 (3.5, 5.2)	1.2E-23	4.4 (3.4, 5.3)	3.6E-20	1.4 (0.9, 1.9)	8.0E-08	1.5 (0.9, 2.0)	1.7E-08	1.5 (0.9, 2.0)	1.1E-07
**BMI, kg/m^2^**	0.2 (–0.3, 0.6)	0.44	0.2 (–0.3, 0.6)	0.43	–0.2 (–0.6, 0.3)	0.54	2.3 (2.1, 2.6)	<1.0E-300	2.3 (2.0, 2.5)	<1.0E-300	1.9 (1.6, 2.2)	8.8E-38
**Waist circumference index, cm/m^2^**	0.2 (–0.2, 0.6)	0.38	0.2 (–0.3, 0.6)	0.42	–0.2 (–0.7, 0.3)	0.49	2.2 (1.9, 2.5)	<1.0E-300	2.1 (1.9, 2.4)	<1.0E-300	1.8 (1.5, 2.1)	9.3E-31
**Waist-hip ratio**	–0.1 (–0.7, 0.4)	0.66	–0.2 (–0.8, 0.4)	0.50	–0.5 (–1.2, 0.1)	0.09	2.2 (1.9, 2.5)	3.4E-37	2.1 (1.8, 2.4)	6.2E-34	1.7 (1.3, 2.1)	2.4E-19
**Total fat %**	0.1 (–0.5, 0.7)	0.71	0.1 (–0.5, 0.7)	0.70	–0.3 (–0.9, 0.3)	0.34	2.6 (2.2, 2.9)	<1.0E-300	2.5 (2.2, 2.9)	<1.0E-300	2.0 (1.6, 2.4)	6.3E-25
**Trunk fat %**	0.0 (–0.4, 0.5)	0.93	0.0 (–0.4, 0.5)	0.90	–0.3 (–0.8, 0.2)	0.25	1.9 (1.6, 2.2)	<1.0E-300	1.9 (1.6, 2.1)	1.6E-37	1.4 (1.1, 1.7)	9.8E-20
**Total fat mass index, kg/m^2^**	0.2 (–0.3, 0.7)	0.38	0.2 (–0.3, 0.7)	0.37	–0.2 (–0.8, 0.3)	0.44	2.5 (2.2, 2.7)	<1.0E-300	2.4 (2.1, 2.7)	<1.0E-300	2.0 (1.7, 2.3)	8.4E-34
**Trunk fat mass index, kg/m^2^**	0.1 (–0.3, 0.6)	0.56	0.1 (–0.3, 0.6)	0.55	–0.2 (–0.7, 0.3)	0.35	2.1 (1.9, 2.4)	<1.0E-300	2.1 (1.8, 2.4)	<1.0E-300	1.7 (1.4, 2.0)	2.3E-29
**Total fat free mass index, kg/m^2^**	0.2 (–0.4, 0.8)	0.55	0.2 (–0.4, 0.8)	0.54	–0.1 (–0.8, 0.6)	0.84	2.8 (2.4, 3.1)	<1.0E-300	2.7 (2.3, 3.1)	<1.0E-300	2.3 (1.9, 2.7)	6.9E-26
**Trunk fat free mass index, kg/m^2^**	0.1 (–0.5, 0.7)	0.80	0.1 (–0.5, 0.7)	0.81	–0.1 (–0.8, 0.6)	0.73	2.3 (1.9, 2.7)	1.85E-34	2.3 (1.9, 2.6)	6.4E-33	2.0 (1.5, 2.4)	2.5E-20

Model 1: multilevel model adjusts for age, sex, ethnicity, and UK Biobank center as fixed effects and a random effect for person to allow for repeated images within person (*n* = 54,714). Model 2 adjusts as model 1 plus BMI, smoking, and Townsend deprivation index as fixed effects (*n* = 54,714). Model 3 adjusts as model 2 but excludes those with diabetes, heart attack, or stroke; those on medication for blood pressure; or those who answered unknown or declined to answer (*n* = 45,644). For body composition measures, percentage difference in vessel tortuosity is per standard deviation increase.

**Table 3:** Adjusted mean difference in vessel diameter in microns with age, sex and per standard deviation increase in body composition measures

Risk marker	Absolute difference in arteriolar diameter (95% CI) in μm						Absolute difference in venular diameter (95% CI) in μm					
	Model 1		Model 2		Model 3		Model 1		Model 2		Model 3	
		*P*		*P*		*P*		*P*		*P*		*P*
**Age per decade**	–0.6 (–0.6, –0.5)	<1.0E-300	–0.5 (–0.6, –0.4)	<1.0E-300	–0.6 (–0.6, –0.5)	6.8E-37	1.0 (0.9, 1.1)	<1.0E-300	1.1 (0.9, 1.2)	<1.0E-300	1.0 (0.9, 1.2)	<1.0E-300
**Female versus male**	–0.2 (–0.3,–0.1)	0.005	–0.1 (–0.3, 0.0)	0.04	0.0 (–0.2, 0.1)	5.5E-01	–0.7 (–0.9, –0.5)	7.1E-11	–0.6 (–0.8, –0.4)	1.6E-08	–0.6 (–0.9, –0.4)	1.4E-07
**BMI,kg/m** ^**2**^	–0.3 (–0.3,–0.2)	1.03E-17	–0.3 (–0.3,–0.2)	5.60E-17	–0.3 (–0.4, –0.3)	9.5E-19	0.6 (0.5, 0.7)	6.3E-25	0.6 (0.5, 0.7)	9.5E-26	0.6 (0.5, 0.7)	8.2E-21
**Waist circumference index, cm/m^2^**	–0.5 (–0.6, –0.4)	<1.0E-300	–0.5 (–0.6, –0.5)	<1.0E-300	–0.6 (–0.7, –0.5)	<1.0E-300	0.4 (0.3, 0.5)	4.8E-13	0.4 (0.3, 0.5)	7.7E-12	0.4 (0.2, 0.5)	3.4E-08
**Waist-hip ratio**	–0.3 (–0.4, –0.3)	2.3E-15	–0.4 (–0.5, –0.3)	1.9E-18	–0.5 (–0.6, –0.4)	3.3E-22	0.5 (0.4, 0.7)	2.7E-13	0.5 (0.3, 0.6)	2.8E-10	0.4 (0.3, 0.6)	3.9E-07
**Total fat %**	–0.3 (–0.4, –0.2)	1.6E-09	–0.3 (–0.3,–0.2)	7.0E-09	–0.3 (–0.4, –0.2)	1.8E-10	0.8 (0.7, 1.0)	2.0E-27	0.8 (0.7, 1.0)	1.9E-28	0.8 (0.7, 1.0)	1.1E-23
**Trunk fat %**	–0.1 (–0.2,–0.1)	1.9E-04	–0.1 (–0.2,–0.1)	6.9E-04	–0.2 (–0.2,–0.1)	3.8E-05	0.7 (0.6, 0.8)	2.1E-28	0.7 (0.6, 0.8)	6.4E-30	0.7 (0.6, 0.8)	8.5E-25
**Total fat mass index, kg/m^2^**	–0.2 (–0.3,–0.2)	3.8E-11	–0.2 (–0.3,–0.2)	1.4E-10	–0.3 (–0.4, –0.2)	1.8E-12	0.7 (0.5, 0.8)	2.7E-27	0.7 (0.6, 0.8)	3.7E-28	0.7 (0.6, 0.8)	4.5E-23
**Trunk fat mass index, kg/m^2^**	–0.2 (–0.2,–0.1)	3.7E-07	–0.2 (–0.2,–0.1)	1.5E-06	–0.2 (–0.3,–0.1)	4.0E-08	0.6 (0.5, 0.8)	1.5E-29	0.7 (0.5, 0.8)	7.9E-31	0.7 (0.5, 0.8)	2.4E-25
**Total fat free mass index, kg/m^2^**	–0.5 (–0.6, –0.4)	1.3E-27	–0.5 (–0.6,–0.4)	1.2E-26	–0.6 (–0.7, –0.5)	1.7E-25	0.5 (0.4, 0.7)	1.3E-10	0.5 (0.4, 0.7)	3.9E-11	0.5 (0.3, 0.7)	1.7E-08
**Trunk fat free mass index, kg/m^2^**	–0.5 (–0.6, –0.4)	2.2E-30	–0.5 (–0.6, –0.4)	7.5E-30	–0.6 (–0.7, –0.5)	2.1E-26	0.3 (0.2, 0.5)	5.3E-05	0.3 (0.2, 0.5)	4.1E-05	0.3 (0.1, 0.5)	0.001

Model 1: multilevel model adjusts for age, sex, ethnicity and UKBB centre as fixed effects and a random effect for person to allow for repeated images within person (*n* = 54,714). Model 2 adjust as model 1 plus body mass index, smoking and Townsend deprivation index as fixed effects (*n* = 54,714. Model 3 adjusts as Model 2 but excludes those with diabetes, heart attack, stroke, on medication for blood pressure or who answered unknown or declined to answer (*n* = 45,644). For body composition measures absolute difference in vessel width is per standard deviation increase.

## Study Importance

**What is already known?**

▶ The microvasculature has increasingly been recognized as an early determinant of both obesity and cardiovascular disease risk.
▶ Research has observed that higher BMI is associated with wider and more tortuous venules and narrower and less tortuous arterioles.

**What does this study add?**

▶ Different attributes of the retinal microvasculature could play distinct roles in cardiometabolic disease prediction.
▶ Venular tortuosity and diameter are more influenced by adiposity, whereas arteriolar diameter relates more strongly to fat-free mass.

**How might these results change the direction of research or the focus of clinical practice?**

▶ Understanding the distinct associations of adiposity and body composition with retinal microvascular changes may improve cardiometabolic disease prediction.
