# Supplementary material for: Retinal Vascular Tortuosity and Diameter Associations with Adiposity and Components of Body Composition
Source: Obesity (Silver Spring). Author manuscript; Available in PMC 2021 Jan 28. (PMC7116641; doi:10.1002/oby.22885)
Supplement: Supplementary file 1 [file EMS112131-supplement-Supplementary_Table_S1.docx]

Table S1 Adjusted differences in vessel tortuosity and diameter per SD change in body composition measure with additional adjustment for total fat free mass index

	Percentage Difference Tortuosity (95%CI)				Difference in Diameter (95%CI)			
**Risk marker**	**Arterioles**	**pvalue**	**Venules**	**pvalue**	**Arterioles**	**pvalue**	**Venules**	**pvalue**
**BMI Kg/m²**	0.25 (-0.58, 1.10)	0.555	2.54 (2.02, 3.05)	**1.1E-22**	0.07 (-0.05, 0.20)	0.257	0.99 (0.77, 1.21)	**3.0E-19**
Total fat free mass index kg/m²	-0.11 (-1.31, 1.09)	0.851	-0.40 (-1.12, 0.32)	0.272	-0.60 (-0.78, -0.42)	**9.3E-11**	-0.68 (-0.99, -0.37)	**1.9E-05**
**Waist circumference index cm/m²**	0.16 (-0.45, 0.77)	0.611	1.54 (1.17, 1.91)	**1.3E-16**	-0.54 (-0.63, -0.45)	**1.0E-30**	0.23 (0.07, 0.39)	0.004
Total fat free mass index kg/m²	0.04 (-0.81, 0.90)	0.927	1.21 (0.70, 1.73)	**3.6E-06**	0.01 (-0.12, 0.14)	0.899	0.31 (0.09, 0.53)	**0.005**
**Waist hip ratio**	-0.28 (-0.90, 0.34)	0.370	1.31 (0.93, 1.69)	**6.2E-12**	-0.25 (-0.34, -0.15)	**2.8E-07**	0.33 (0.17, 0.49)	**6.6E-05**
Total fat free mass index kg/m²	0.32 (-0.36, 0.99)	0.361	2.14 (1.73, 2.55)	**1.0E-24**	-0.40 (-0.51, -0.30)	**8.8E-15**	0.39 (0.22, 0.57)	**9.5E-06**
**Total fat %**	0.03 (-0.62, 0.68)	0.934	1.71 (1.31, 2.10)	**1.3E-17**	-0.04 (-0.14, 0.06)	0.461	0.77 (0.61, 0.94)	**1.6E-19**
Total fat free mass index kg/m²	0.19 (-0.52, 0.90)	0.599	1.81 (1.38, 2.24)	**8.7E-17**	-0.49 (-0.60, -0.39)	**1.5E-19**	0.14 (-0.04, 0.32)	0.137
**Trunk fat %**	0.02 (-0.46, 0.50)	0.941	1.54 (1.25, 1.83)	**2.7E-25**	-0.03 (-0.11, 0.04)	0.383	0.68 (0.55, 0.80)	**8.6E-27**
Total fat free mass index kg/m²	0.07 (-0.56, 0.70)	0.824	1.80 (1.42, 2.19)	**1.1E-20**	-0.53 (-0.62, -0.43)	**1.7E-27**	0.13 (-0.03, 0.29)	0.111
**Total fat mass index kg/m²**	0.21 (-0.43, 0.86)	0.513	1.92 (1.53, 2.31)	**2.2E-22**	0.06 (-0.04, 0.15)	0.245	0.76 (0.59, 0.92)	**2.1E-19**
Total fat free mass index kg/m²	0.00 (-0.84, 0.85)	0.999	0.96 (0.45, 1.47)	**2.2E-04**	-0.56 (-0.69, -0.44)	**6.1E-18**	-0.14 (-0.36, 0.08)	**0.200**
**Trunk fat mass index kg/m²**	0.14 (-0.35, 0.63)	0.585	1.72 (1.42, 2.02)	**3.0E-30**	0.01 (-0.06, 0.09)	0.716	0.70 (0.57, 0.82)	**1.7E-27**
Total fat free mass index kg/m²	-0.01 (-0.69, 0.68)	0.977	1.16 (0.74, 1.58)	**3.9E-08**	-0.54 (-0.65, -0.44)	**8.3E-25**	-0.11 (-0.29, 0.06)	**0.209**

Adiposity measures are presented as z scores. Body mass index (BMI), confidence interval (CI). Regression coefficients are from a multilevel model allowing for repeated images from the same person (random effect for person). Models adjusting each factor for age, sex, ethnicity, UKBB centre, smoking and the Townsend deprivation index and fat free mass index as fixed effects (n=54,714).
